# The bacterial chromosome: architecture and action of bacterial SMC and SMC-like complexes

**DOI:** 10.1111/1574-6976.12045

**Published:** 2013-11-18

**Authors:** Sophie Nolivos, David Sherratt

**Affiliations:** Department of Biochemistry, University of OxfordOxford, UK

**Keywords:** SMC, chromosome, condensin, cohesin, chromosome organization, chromosome segregation

## Abstract

Structural Maintenance of Chromosomes (SMC) protein complexes are found in all three domains of life. They are characterized by a distinctive and conserved architecture in which a globular ATPase ‘head’ domain is formed by the N- and C-terminal regions of the SMC protein coming together, with a *c*. 50-nm-long antiparallel coiled-coil separating the head from a dimerization ‘hinge’. Dimerization gives both V- and O-shaped SMC dimers. The distinctive architecture points to a conserved biochemical mechanism of action. However, the details of this mechanism are incomplete, and the precise ways in which this mechanism leads to the biological functions of these complexes in chromosome organization and processing remain unclear. In this review, we introduce the properties of bacterial SMC complexes, compare them with eukaryotic complexes and discuss how their likely biochemical action relates to their roles in chromosome organization and segregation.

## Identification, initial characterization and distribution of Structural Maintenance of Chromosomes (SMC) complexes

**Fig. 1:**
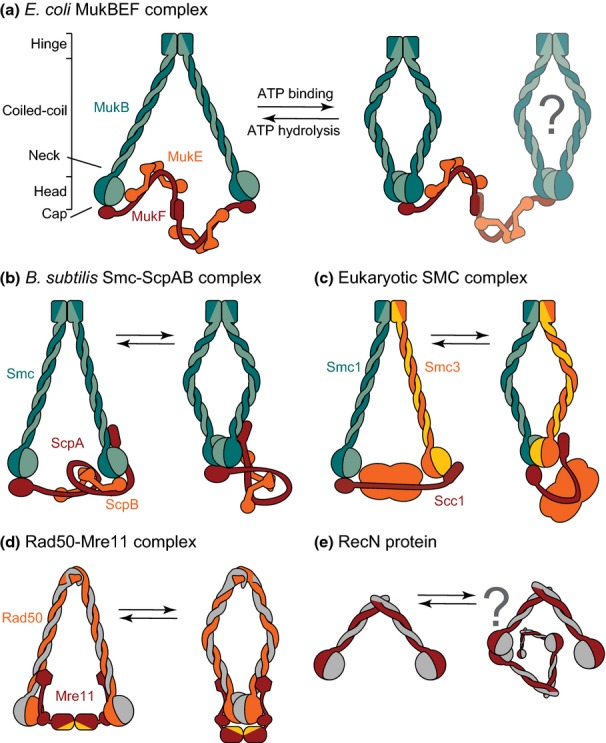
Architecture of SMC complexes. SMC proteins are composed of three distinctive parts, a head ATPase domain formed from the SMC N- and C-termini, a long intramolecular coiled-coil and a hinge dimerization domain. The complex is formed by an SMC dimer bridged by a kleisin (brown) associated with a second non-SMC subunit (orange). (a) The C-terminal domain of *Escherichia coli* MukF interacts with the ‘cap’ region of the MukB head, while the central region interacts with a homodimer of MukE. In the absence of ATP, two MukF and four MukE bind a dimer of MukB. ATP binding displaces one MukF giving a stoichiometry similar to other SMC complexes. However, a dimer of dimers can also be potentially formed via dimerization of the MukF N-terminal domain. (b) In *Bacillus subtilis*, the C-terminal domain of the kleisin ScpA interacts with the cap region of one Smc head, and the N-terminus binds the ‘neck’ region (interface between the coiled-coil domain and the head) of the other Smc monomer. In addition, a central ScpA domain wraps around a dimer of ScpB. ATP binding and head engagement prevent a second ScpA binding. (c) The core of eukaryotic SMC complexes is composed of an SMC heterodimer, a kleisin and at least one other non-SMC subunit. In addition, several other accessory proteins are required during SMC complex action. The cohesin complex is illustrated here. For clarity, an orange cloud represents non-SMC subunits. The C-terminal domain of the kleisin Scc1 interacts with the cap domain of Smc1, whereas the N-terminal domain interacts with the head domain of Smc3. A putative ‘neck’ interaction is shown, as in *B. subtilis*. (d) The Rad50-Mre11 complex is symmetrical in both ATP-bound and unbound forms. SMC-like protein Rad50 differs from true SMC proteins by having a zinc–hook dimerization domain. Dimerization is thought to be weak. The Mre11 C-terminal helix-loop-helix domain, represented by a hexagon, binds the Rad50 neck, while the central ‘capping’ domain interacts with the head. The N-terminal dimerization domain carries DNA binding and nuclease activities (yellow). ATP-dependent head engagement causes a dramatic rearrangement within the complex, suggesting a rotation of the coiled-coil domain. Structures of the complex were obtained with only a short part of the coiled-coil, and it is not clear whether this rearrangement would be possible in a full-length dimeric protein as drawn here or whether head engagement breaks the zinc–hook interaction. (e) RecN is an SMC-like protein that acts in DSB repair without known accessory proteins and possesses a short coiled-coil of about one quarter of the length of other SMC proteins. The dimerization interface is contained in the three apical α-helices. The rigidity of the coiled-coil is proposed to disfavour head engagement within a dimer, but to favour interactions between two dimers, potentially allowing ATP-dependent polymerization along the DNA.

Our story starts with the isolation of an *Escherichia coli mukB* mutant (from the Japanese word ‘Mukaku’, meaning ‘anucleate’) that generated anucleate cells as a consequence of impaired chromosome segregation, by Soto Hiraga, a pioneer of bacterial chromosome biology (Hiraga *et al*., 1989). Subsequent analysis characterized the *mukB* gene and its 177-kDa protein, MukB, which was shown to form V-shaped dimers in which the C- and N-terminal regions came together to form a globular ATP-binding ‘head’, with a rod-like central domain and a distant ‘hinge’ (Fig. 1; Niki *et al*., 1991, 1992). It was proposed that this might be a force-generating motor-like protein, akin to myosin, kinesin or dynein, which functioned directly in chromosome segregation. Later, the *E. coli mukB* gene was shown to be in an operon in which two other genes, *mukE* and *mukF,* encoded proteins necessary for Muk function, whereas the first gene of the operon encodes a presumptive methyl transferease, SmtA, of unknown function (Yamanaka *et al*., 1996). Other bacteria that encode *mukBEF* genes also have them organized into a single operon, whereas most other bacterial SMC proteins are encoded in an operon distinct from those of their cognate accessory proteins, which themselves tend to be cotranslationally expressed from within the same operon.

In 1993, 1993Strunnikov *et al*., characterized a yeast gene, *SMC1*, and its product, Smc1p, identified earlier by a mutant phenotype of impaired minichromosome segregation (Larionov *et al*., 1985). This essential protein had the same general organization of a central coiled-coil ‘rod’, bounded by a dimerization domain and an ATPase formed by the N- and C-termini, as in MukB, and the SMC-like Rad50 protein, involved in DNA repair (Alani *et al*., 1989). Furthermore, Strunnikov *et al*. (1993) identified putative proteins related to Smc1p from bioinformatic searches in a number of other eubacteria. Melby *et al*. (1998) extended the bioinformatic and phylogenetic analysis and defined MukB as a *bona fide,* yet distant, SMC family member. In addition, their biochemical and electron microscopy analysis led them to articulate clearly the distinctive and conserved architecture of SMC V-shaped dimers in which a *c*. 50-nm-long antiparallel intramolecular coiled-coil folded about a flexible dimerization hinge, with the ATPase head domain created by the SMC N- and C-termini at the opposite end to the hinge (discussed in Haering *et al*., 2002). Some O-shaped dimers arose by interaction between the two heads of a dimer.

Later, it became clear that SMC proteins in general do not function in isolation, and like MukB, they interact with essential accessory proteins (Fig. 1; Michaelis *et al*., 1997; Yamazoe *et al*., 1999; Mascarenhas *et al*., 2002; Nasmyth & Haering, 2005). One of these, termed ‘the kleisin’, bridges the two ATPase heads of an SMC dimer and interacts with a second accessory protein. Together, these play key regulatory roles in the ATP binding–hydrolysis–release cycles. Eukaryotic SMCs invariably form heterodimers, with the N- and C-terminal domains of the kleisin interacting with specific SMC heads (Fig. 1; Haering *et al*., 2002; Onn *et al*., 2007). In contrast, bacterial SMCs are invariably homodimers. In eukaryotes, SMC complexes have been classified into three groups, cohesins, condensins and Smc5-6 complexes. Classically, cohesins maintain cohesion between replicated sister chromatids by entrapping the sisters within a tripartite SMC complex ring, while condensins act in condensation–organization of mitotic chromosomes and Smc5-6 complexes act in DNA repair (Thadani *et al*., 2012; Wu & Yu, 2012; Remeseiro & Losada, 2013). Furthermore, all of these complexes have been implicated directly or indirectly in other chromosome processing activities. Typically, bacterial SMC complexes have been described as condensins although it remains unclear as to whether their primary role is in chromosome condensation. Certainly, *E. co*li MukBEF is not a cohesin because the newly replicated sister chromosomes of Muk^−^ cells have increased rather than decreased cohesion (Danilova *et al*., 2007). SMC protein complexes are ubiquitous and, as far as we are aware, are present in all organisms except a few bacteria.

Until recently, a single characteristic SMC complex was thought to be encoded by any given bacterium. For example, the Gm^+^ bacterium *Bacillus subtili*s and Gm^−^ alphaproteobacterium *Caulobacter crescentus* encode a typical SMC protein, a kleisin ScpA (Segregation and condensation protein A), and ScpB, which binds ScpA (Britton *et al*., 1998; Melby *et al*., 1998; Jensen & Shapiro, 1999; Mascarenhas *et al*., 2002; Bürmann *et al*., 2013). The *E. coli* SMC protein, MukB, and its accessory proteins, MukE and MukF (kleisin), have a low primary sequence homology to the more typical SMC complexes. MukBEF complexes are restricted to some *Gamma*- and *Deltaproteobacteria*, which additionally contain a number of restricted and characteristic genes that include *dam*, *seqA*, *mutH*, *matP* and *zapB* and a number of other genes of unknown function (Hiraga, 2000; Brézellec *et al*., 2006). The significance of this cluster of genes remains to be elucidated, but it is intriguing that those characterized all participate in chromosome organization processing, and mutations in at least two of these genes suppress some of the phenotypes of *muk* mutations (Onogi *et al*., 2000). A third bacterial SMC complex was identified recently through bioinformatic analysis and has been termed ‘MksBEF’ for MukBEF-like SMC (Petrushenko *et al*., 2011). Unlike MukBEF, these genes are widely distributed in bacterial species and often found in combination with MukBEF, SMC-ScpAB and/or other MksBEF complexes. MksB has a shorter coiled-coil than typical SMCs, and its precise functions remain unclear.

In addition to true SMC complexes, a family of conserved SMC-like proteins that include the related bacterial SbcC and archaeal and eukaryotic Rad50 has important roles in DNA repair at DSB ends; A *c*. 50-nm antiparallel coiled-coil is bounded by a zinc-hook dimerization domain, and the typical ATPase head is bridged by the nuclease SbcD/Mre11 (Fig. 1; Lammens *et al*., 2011; Lim *et al*., 2011; Möckel *et al*., 2012; Stracker & Petrini, 2011). Finally, another SMC-like bacterial repair–recombination protein, RecN, has been proposed also to act in DSB repair, although atypically no accessory proteins appear to be involved in its action, and its coiled-coil is much shorter than the typical *c*. 50 nm (Pellegrino *et al*., 2012).

## Insight into bacterial SMC complex function from genetic studies

Mutations in SMC/MukB (hereafter termed ‘SMC’ for brevity), or in either of the accessory proteins, lead to the same phenotype, showing that all three proteins are required for SMC complex function. Impairment of SMC function generally leads to an increase in the production of anucleate cells, indicating a direct or indirect role in chromosome segregation (Table 1). In *C. crescentus*, *B. subtilis* and *E. coli*, this is accompanied by temperature sensitivity, which is particularly acute under fast growth conditions. Furthermore, global chromosome decondensation has been reported (Niki *et al*., 1991; Moriya *et al*., 1998; Britton *et al*., 1998; Jensen & Shapiro, 1999; Sawitzke & Austin, 2000; Table 1). These defects are frequently enhanced under conditions that lead to decreased negative supercoiling (gyrase inhibitors or appropriate gyrase mutations; Hiraga *et al*., 1991; Britton *et al*., 1998; Moriya *et al*., 1998; Jensen & Shapiro, 1999; Lindow *et al*., 2002; Adachi & Hiraga, 2003), while conditions that increase negative supercoiling can suppress the temperature-sensitive phenotype resulting from Muk/SMC impairment (for example, TopA impairment in *E. coli* and *B. subtilis*; Sawitzke & Austin, 2000; Lindow *et al*., 2002).

**Table 1:** Phenotypes associated with mutation in genes of bacterial SMC complexes

	Deletions	T.S.	Anucleate cells	Disorg.	Decond.	Gyrase inh.	Cell morphology	References
*Alpha-proteobacteria*								
*C. crescentus* SMC-ScpAB	Δ*smc*	Yes	0.1% in p. and n.p.	10–15%	Yes	n.d.	Cell cycle arrest at predivisional stage	Jensen & Shapiro (1999, 2003) and Schwartz & Shapiro (2011)
*Gamma-proteobacteria*								
*E. coli* MukBEF	Δ*mukB*	Yes	10–20% in n.p., 5% in p.	Yes	Yes	Yes	Filament	Niki *et al*. (1991), Yamanaka *et al*. (1996), Sawitzke & Austin (2000) and Danilova *et al*. (2007)
*P. aeruginosa* SMC-ScpAB	Δ*smc*	No	2.5%	n.d.	n.d.	n.d.	WT	Petrushenko *et al*. (2011)
*P. aeruginosa* MksBEF	Δ*mksB*	No	1.2% only in M9 at 37 °C	n.d.	n.d.	n.d.	WT	Petrushenko *et al*. (2011)
*Firmicutes*								
*B. subtilis* SMC-ScpAB	Δ*smc*	Yes	10% in p., >10% in n.p.	Yes	Yes	Yes	Elongated cells	Britton *et al*. (1998), Moriya *et al*. (1998) and Lindow *et al*. (2002)
*B. subtilis* SMC-ScpAB	Δ*smc*Δ*spo0J*	Yes	26% in p.	Yes	Yes	n.d.	Elongated cells	Britton *et al*. (1998)
*S. pneumoniae* SMC-ScpAB	Δ*smc*	No	0.5% at 30 °C, 1.8% at 37 °C	n.d.	No	No	WT	Minnen *et al*. (2011)
*S. aureus* SMC-ScpAB	Δ*smc*	No	10% at 30 °C, 8% at 37 °C and 2.5% at 42 °C	n.d.	No	n.d.	WT	Yu *et al*. (2010)
*S. aureus* SMC-ScpAB	Δ*smc*Δ*spoIIIE*	Yes	4% in p., 6% in n.p.	Yes	n.d.	n.d.	Heterogeneous	Yu *et al*. (2010)
*Actinobacteria*								
*S. coelicolor* SMC-ScpAB	Δ*smc*	No	7%	n.d.	Yes	n.d.	WT	Dedrick *et al*. (2009) and Kois *et al*. (2009)
*S. coelicolor* SMC-ScpAB	Δ*smc*Δ*parB*	No	23%	n.d.	n.d.	n.d.	Spore compartments with altered shape and size	Dedrick *et al*. (2009), Kois *et al*. (2009)
*M. smegmatis* SMC-ScpAB	Δ*smc*	No	n.d.	n.d.	n.d.	No	n.d.	Güthlein *et al*. (2008)
*M. tuberculosis* SMC-ScpAB	Δ*smc*	No	n.d.	n.d.	n.d.	n.d.	n.d.	Güthlein *et al*. (2008)
*Deinococcus-thermus*								
*D. radiodurans* SMC-ScpB	Δ*smc*	No	No	No	No	Yes	WT	Bouthier de la Tour *et al*. (2009)
*Archaea euryarchaeot*								
*M. voltae* SMC-ScpAB	*smc::pac*	No	20%	n.d.	No	n.d.	1–2% titan cells	Long & Faguy (2004)

Anucleate cells: percentage of anucleate cells (or spores in the case of *S. coelicolor*) in the conditions indicated.

T.S., temperature-sensitive growth; Disorg, altered nucleoid organization; Decond, decondensation of the nucleoid; Gyrase inh, hypersensitivity to gyrase inhibitor; Cell morphology, after a switch to nonpermissive growth conditions when applicable; p, permissive growth conditions; n.p, after a switch to nonpermissive growth; onditions; n.d, not determined; WT, wild type.

If SMC complexes have a direct role in chromosome segregation, then in their absence, what other mechanisms mediate or facilitate chromosome segregation? Two other systems that act in chromosome segregation are the ParAB–*parS* systems that are best characterized for their roles in low copy plasmid segregation and the FtsK/SpoIIIE family of DNA translocases (Kaimer & Graumann, 2011; Mierzejewska & Jagura-Burdzy, 2012; Reyes-Lamothe *et al*., 2012). Mutation of either of these systems does not generally lead to a loss of cell viability (an exception being loss of functional ParAB in *C. crescentus*; Mohl & Gober, 1997), but can be synthetically lethal or sick in combination with SMC impairment. For example, in *Staphylococcus aureus* and *Streptomyces coelicolor*, the double mutants Δ*smc*Δ*spoIIIE* and Δ*smc*Δ*parB,* respectively, are viable, but have phenotypes considerably stronger than the relatively mild phenotypes of the single *parB* and *spoIIIE* mutants (Dedrick *et al*., 2009; Yu *et al*., 2010). When the Muk^−^/SMC^−^ phenotype is strong, the phenotypes of some double combinations are synthetically lethal, for example, an *E. coli mukB-ftsK*_*C*_ double mutant (Yu *et al*., 1998; Sivanathan *et al*., 2009) and the double mutants in SMC-SpoIIIE, SMC-SftA (an FtsK orthologue) and SMC-Soj/Spo0J in *B. subtilis* (Britton *et al*., 1998; Britton & Grossman, 1999; Lee & Grossman, 2006; Kaimer *et al*., 2009). The overlapping activities of multiple SMC complexes may account for some weak SMC^−^ phenotypes like in *Pseudomonas aeruginosa* (Petrushenko *et al*., 2011).

Analysis of mutant phenotypes as outlined above has failed to determine conclusively whether the primary role of SMC/MukB complexes is in chromosome organization, which would reasonably require that the SMC complex interacts globally with different regions of the chromosome, or whether the primary role is in chromosome segregation as proposed in the original experiments of Hiraga, Niki and colleagues. In the latter case, one would expect the SMC complex to interact with a specific chromosomal region. A third possibility is that these complexes act independently in both organization and segregation. If this is the case, it should be possible to separate these functions genetically. A limitation of these classical genetic studies results from the strong pleiotropic phenotypes that arise from mutation in Muk/SMC, ParAB-*parS* or FtsK and its orthologs. Quantitative cell biology studies combined with real-time depletion and repletion experiments have begun to address this (later).

## Architectural features and biochemical activities

**Fig. 2:**
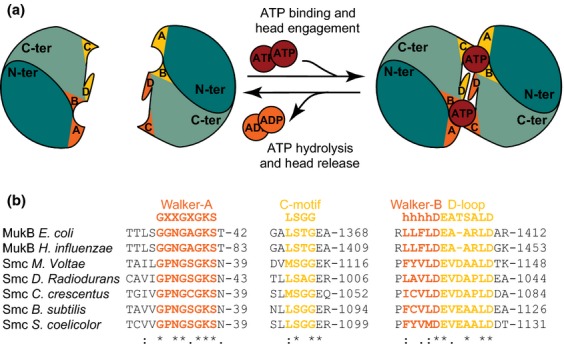
Conservation of ATPase head domains. (a) Head engagement in an SMC dimer forms two ATPase domains, indicated in yellow and orange. Appropriate letters specify the four conserved motifs. For each ATPase domain, the WalkerA and WalkerB motifs are carried, respectively, by the N-terminal (in dark green) and the C-terminal parts (in light green) of one SMC protein, whereas the C-motif and D-loop are found in the C-terminal domain of the second SMC monomer. ATP hydrolysis leads to head disengagement. (b) Alignment, using Clustal Omega (Goujon *et al*., 2010), showing the conservation of the four characteristic motifs among bacterial SMC proteins. ‘*’ indicates positions that have a single, fully conserved residue; ‘:’ and ‘.’ indicate conservation between groups of strongly and weakly similar properties, respectively. Consensus sequences are indicated. NCBI accession numbers are as follows: BAA06510.1, YP_249221.1, YP_003707593.1, CAD10418.1, AAF00713.1, NP_389476.2, NP_629712.1.

The SMC ATPase head domain is a characteristic ABC transporter ATPase, characterized by Walker A and Walker B consensus motifs (Walker *et al*., 1982), the signature motifs or C-motif unique to the ABC superfamily (Ames & Lecar, 1992) and a D-loop (Fig. 2). The Walker A motif, which is essential for ATP binding, is encoded by the SMC N-terminus, whereas the Walker B motif, required for ATP hydrolysis, is encoded by the C-terminus, with the N- and C-termini folding into a compact globular domain. Within an SMC dimer, the C-terminal domain of the second monomer carries the C- or signature motif and the D-loop that stabilize the binding of ATP by the first monomer, and is required for ATP hydrolysis. Consequently, two composite ATP-binding sites are created by dimerization of the two head domains (Fig. 2). ATP binding leads to head engagement, resulting in the formation of the ring-shaped SMC dimer in which the kleisin association with both heads in a dimer reinforces the O-shaped structure by forming an SMC–kleisin tripartite ring (or a MukB-MukF tetrapartite ring in the case of MukBEF; Fig. 1; Woo *et al*., 2009; Bürmann *et al*., 2013; reviewed in Hirano & Hirano, 2006; Lim & Oh, 2009). ATP binding and head engagement are invariably required for stable association with DNA, while ATP hydrolysis, which can only occur when the heads are engaged, is required for normal function (Hirano & Hirano, 2006; Schwartz & Shapiro, 2011; Badrinarayanan *et al*., 2012b; Bürmann *et al*., 2013). *In vitro* ATPase assays of MukBEF have shown that robust ATP hydrolysis can occur in the absence of added DNA, but that requires the presence of both MukE and MukF in addition to MukB (Woo *et al*., 2009; reviewed in Lim & Oh, 2009) Similarly, *B. subtilis* SMC ATPase activity is stimulated by accessory proteins (Kamada *et al*., 2013). Note that both ATP binding and kleisin association with the heads act to keep the heads within a dimer ‘closed’.

Eukaryotic SMC complexes are intrinsically asymmetric because they are formed from SMC heterodimers. Despite bacterial complexes containing SMC homodimers, asymmetric complexes form at least during part of the ATP binding–hydrolysis–release cycles, indicating that asymmetry underlies their conserved biochemical action. An analysis that focussed on the *B. subtilis* SMC complex showed that a kleisin monomer bridges the two heads of an SMC homodimer, thereby introducing asymmetry into the tripartite ring-shaped complex (Bürmann *et al*., 2013). The kleisin C-terminus interacts with the ‘cap’ region of one head, while the N-terminal domain interacts with the SMC ‘neck’, a region of the coiled-coil adjacent to the ATPase (Fig. 1). This asymmetric interaction is facilitated during head engagement resulting from ATP binding, because a steric clash prevents the binding of two kleisin C-terminal domains to the two heads of a single SMC homodimer. The architecture of this asymmetric complex is remarkably similar to that proposed for eukaryotic SMC complexes (Bürmann *et al*., 2013; Upton & Sherratt, 2013). At first sight, the architecture of the MukBEF complex looks dissimilar because the kleisin, MukF, dimerizes through its N-terminal domain, while the C-terminal domains interact with a ‘cap’ in the heads of the MukB dimer in the absence of nucleotide, giving a symmetrical complex (Fig. 1; Woo *et al*., 2009). However, ATP binding and consequent head engagement sterically prevent occupation of both heads by kleisin C-terminal domains, thereby generating an asymmetric complex (Fig. 1). This could lead to dimer of dimer complexes in the presence of ATP, as have been observed *in vivo* (Badrinarayanan *et al*., 2012b) and *in vitro* (Petrushenko *et al*., 2006; Woo *et al*., 2009). Intriguingly, in Rad50-Mre11 complexes, Mre11 dimerization is accompanied by both cap and neck interactions with the SMC-like Rad50 (Fig. 1; Lim *et al*., 2011; Lammens *et al*., 2011; Möckel *et al*., 2012), and we speculate that MukF might in addition have a neck interaction, potentially leading to asymmetric head engagement through the kleisin interaction in the presence of ATP and thereby helping close a tripartite ring. The theme of ATP-dependent head engagement leading to steric clashes is continued with Rad50-Mre11, where head engagement occludes the nuclease within Mre11 (Lammens *et al*., 2011; Lim *et al*., 2011; Möckel *et al*., 2012).

Structures of the dimerization hinges of several SMC proteins have demonstrated a strong dimerization interface that contains some conserved features (Haering *et al*., 2002; Ku *et al*., 2010; Li *et al*., 2010; Griese & Hopfner, 2011). Nevertheless, several studies have provided evidence that the dimerization interface must open or change for normal function (Gruber *et al*., 2006; Hu *et al*., 2011; Thadani *et al*., 2012). Indeed, it has been proposed that this interface provides the loading gate for DNA, while the head domains are the exit gate (later; Chan *et al*., 2012; Bürmann *et al*., 2013), thereby providing an analogy to type II topoisomerases that have separate DNA entry and exit gates.

A range of studies has assessed SMC binding to DNA *in vitro* (Volkov *et al*., 2003; Hirano & Hirano, 2004, 2006; Petrushenko *et al*., 2006, 2010; Cui *et al*., 2008; Woo *et al*., 2009; Griese & Hopfner, 2011; Borgmann *et al*., 2013b). Some of these have been in the absence of accessory proteins, and few have used conditions in which DNA-binding requirements mimic *in vivo* loading onto chromosomes. Nevertheless, structural studies of *E. coli* MukBEF and an archaeal SMC identified a conserved flat positively charged region on top of the SMC head that interacts with DNA and is likely to play an important role, possibly once DNA is properly loaded into the tripartite ring. Some assays have also assessed DNA compaction, intermolecular DNA bridging or an ability to restrain DNA topology (Petrushenko *et al*., 2006, 2010; Chen *et al*., 2008; Borgmann *et al*., 2013b), but the physiological significance of these remains unclear, as does the study in which SMC overexpression led to DNA condensation *in vivo* (Volkov *et al*., 2003; Wang *et al*., 2006; Petrushenko *et al*., 2011).

Although the early analyses of Hiraga, Niki and colleagues (Niki *et al*., 1991, 1992) proposed that MukB(EF) might function as a force-generating motor-like myosin, kinesin or dyneins, using DNA as a track rather than actin or tubulin, the analogy may well be mechanistically superficial. Nevertheless, there are at least two similarities. For example, movement of cytoskeletal motors on actin and tubulin tracks bears similarities to how MukBEF has been proposed to move on DNA. The slow turnover of MukBEF dimers of dimers on DNA, as compared to measured ATPase rates *in vitro*, led to the proposal of a ‘rock climber’ model in which release of DNA from one dimer upon head opening, mediated by ATP hydrolysis, would allow potentially reloading of a second DNA segment, while the second dimer remained bound to DNA (Badrinarayanan *et al*., 2012b). Another similarity is that cytoskeletal motors carry cargo bound to sites distant from the ATPase heads, analogous to the observed *in vitro* interaction of topoisomerase IV with the MukB hinge (Hayama & Marians, 2010; Li *et al*., 2010). This indicates that TopoIV is a MukBEF cargo that would allow coordination of decatenation and segregation, consistent with the observation that sister cohesion is increased in Muk^−^ cells (Danilova *et al*., 2007).

**Fig. 3:**
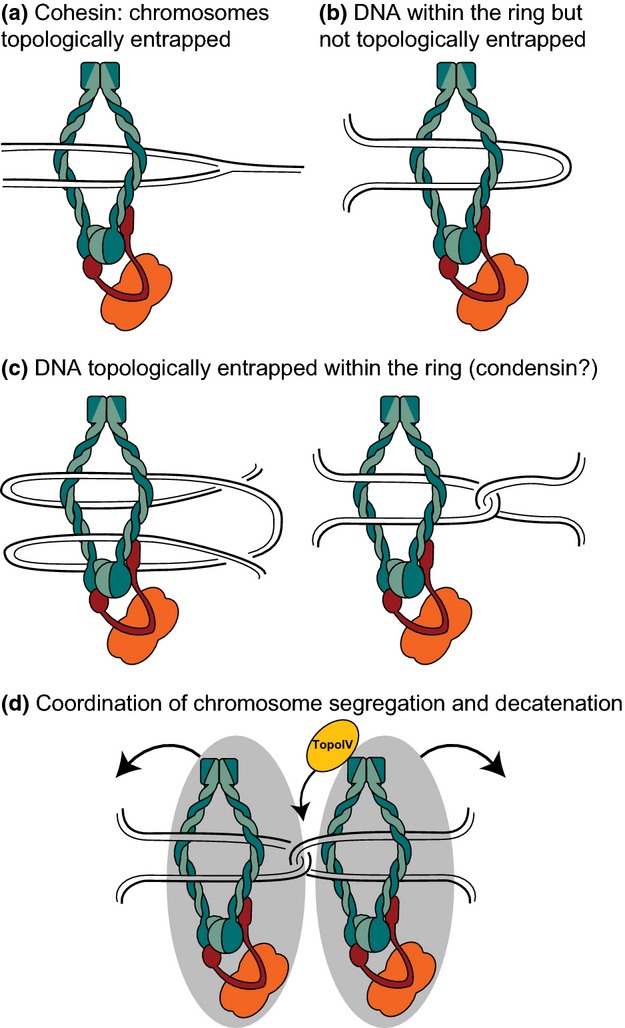
DNA entrapment models. (a) Model for cohesion. The two sister chromatids are topologically entrapped within the cohesin ring. Release of cohesion can occur after kleisin cleavage by separase. (b and c) Models for action as a condensin and for *ori* positioning. Two DNA segments from the same chromosome are enclosed within the ring and can be topologically entrapped (c), or not (b). (d) A model for how bipolar *ori* segregation could be coordinated with sister *ori* decatenation. A cluster of MukBEF complexes (shown as a single complex for simplicity, overlaid on a grey ellipsoid representing the MukBEF cluster) is bound to sister *ori* regions that are catenated immediately after replication (only a single catenation link is shown). If the proposed MukBEF focus positioning system begins to separate to generate two sister MukBEF clusters, action of TopoIV (yellow; associated with the MukBEF clusters) will remove sister chromosome tension and allow sister *ori* separation.

Do all SMC complexes entrap DNA within a closed tripartite ring as shown for cohesin (Haering *et al*., 2002) and supported by experiments with *B. subtilis* Smc (Bürmann *et al*., 2013)? The conserved ability to engage heads on ATP binding and open them on ATP hydrolysis, the closure being reinforced by the kleisin interaction, taken together with observations that led to proposals that ring opening at both the heads and the hinge is necessary for *in vivo* function (Arumugam *et al*., 2003; Weitzer *et al*., 2003; Gruber *et al*., 2006; Chan *et al*., 2012), all support the DNA entrapment model, based on the observed topological entrapment of yeast minichromosomes within cohesin rings (Haering *et al*., 2008; Farcas *et al*., 2011). In the case of cohesin, it is proposed that the two newly replicated sisters are effectively topologically entrapped, because any chromosome ends are distant from a bound SMC complex (Fig. 3). In other situations, for example, where the SMC complex is acting as a condensin, it has been proposed that two duplex segments derived from the same chromosome are contained within a ring. If these were formed from a simple loop of limited size, there would be no topological entrapment, because the loop could be released from (or indeed loaded into) the SMC ring without the ring opening. However, trapping of toroidal links or catenated loops would need ring opening to load or release the DNA (Fig. 3). Despite the strong evidence for tripartite SMC complex rings, *in vitro* experiments have also provided evidence that SMC complexes may ‘daisy-chain’ into oligomeric or rosette structures, and it has been proposed that these may be physiologically relevant (Mascarenhas *et al*., 2005; Matoba *et al*., 2005). Such structures could be mediated simply by kleisin–SMC interactions or through higher-order interactions mediated by other accessory proteins; for example, MukE and *B. subtilis* ScpB have been proposed to be able to mediate such higher-order complexes (Hirano & Hirano, 2004; Gloyd *et al*., 2011). Nevertheless, both *in vivo* and *in vitro* analysis of *B. subtilis* SMC complexes has found predominantly simple dimeric SMC complexes compatible with tripartite rings being the major players in SMC action (Fuentes-Perez *et al*., 2012; Bürmann *et al*., 2013).

Why is the *c*. 50-nm intramolecular coiled-coil a conserved part of SMC complex architecture? We consider two nonexclusive scenarios. In one, the length of the coiled-coils is needed to make a ring that can accommodate chromosomal DNA and anything associated with it; in this, it is simply a long linker that should be relatively immune to mutagenesis. In the other, the coiled-coil is a communication device between the head and hinge; for example, enabling the energy derived from ATP binding–hydrolysis–release of phosphate and/or ADP being used to do work at the hinge (discussed in Thadani *et al*., 2012). In Rad50-Mre11 complexes, the angle of the coiled-coils leaving the heads changes markedly on head engagement (Lammens *et al*., 2011; Lim *et al*., 2011; Möckel *et al*., 2012). In this context, it is intriguing to note that in dynein, energy obtained from the ATP binding–hydrolysis–release cycle is transmitted through the coiled-coil to do work at the distant microtubule-binding domain (the equivalent of the dimerization hinge in SMCs), by changing the register of the two chains in the coiled-coil (Carter, 2013). A similar mechanism could operate within SMCs, with kleisin–neck interactions and other changes associated with the ATPase cycle facilitating this transmission from the head to the hinge.

## Functional insights from cell biology: chromosome organization or segregation; local or global action?

Analysis of chromosome organization in Muk^−^ *E. coli* cells showed that the position of genetic loci changes dramatically in the absence of MukBEF, corresponding to a 90° rotation of the whole chromosome with respect to the long axis of the cell, such that newborn cells growing in minimal medium have their origins at the old pole and replication terminus regions at the new pole (Danilova *et al*., 2007). In such cells, DNA replication now initiates at a pole-proximal origin, showing that origin position defines replisome position at initiation rather than vice versa (Reyes-Lamothe *et al*., 2008). Reorganization of the chromosome after fast Muk repletion and its altered organization on rapid degron-mediated Muk impairment were relatively slow, taking > 30% of a cell generation equivalent for the altered state to be achieved and occurred in the absence of DNA replication (Badrinarayanan *et al*., 2012a).

**Fig. 4:**
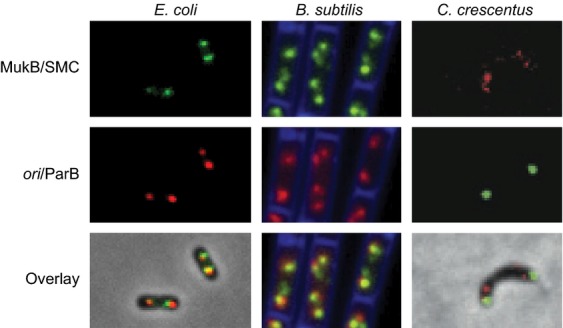
Localization of bacterial SMC. *Escherichia coli* MukB colocalized with the origin of replication and *Bacillus subtilis* SMC foci with Spo0J protein (Sullivan *et al*., 2009). *Caulobacter crescentus* bright foci are located at the cell pole, but colocalized only rarely with ParB proteins (Schwartz & Shapiro, 2011).

The *in vivo* visualization of fluorescent SMC complexes (Table 2) and their relationship with genetic locus position and the replication machinery have shown that *E. coli* MukBEF and other SMC complexes can form a discrete number of fluorescent foci per cell. Niki, Hiraga and colleagues were the first to demonstrate the patterns of MukBEF fluorescent focus positioning in cells (Ohsumi *et al*., 2001). Further analyses showed that MukBEF and *B. subtilis* SMC are *ori*-associated (Fig. 4; Danilova *et al*., 2007; Gruber & Errington, 2009; Sullivan *et al*., 2009; Badrinarayanan *et al*., 2012a) and not associated with functional replisomes. In *C. crescentus*, between two to five foci were observed depending on the cell cycle stage, and one or two bright polar foci were formed only in a minority of late predivisional cells (Jensen & Shapiro, 2003).

**Table 2:** Localization of bacterial SMC

Protein	Growth conditions	Number of foci per cells	Localization	References
*C. crescentus* Smc	MM 28 °C	2–5 foci with 2 bright foci in late predivisional cells	Bright foci are polar, colocalize rarely with ParB	Schwartz & Shapiro (2011)
*E. coli* MukBEF	MM 37 °C	2–4 foci	Colocalize with *oriC*	Badrinarayanan *et al*. (2012a)
*B. subtilis* Smc	MM 30 °C	2–4 foci	Colocalize with Spo0J	Gruber & Errington (2009) and Sullivan *et al*. (2009)
*S. pneumoniae* Smc	RM 30 °C	1–2 foci diffused	Dependent on ParB	Minnen *et al*. (2011)
*S. coelicolor* Smc	MM or RM 30 °C	Punctuated pattern only in predivisional aerial hyphae, not for every nucleoid	Not associated with any particular chromosomal region, SMC foci does not colocalized with ParB	Dedrick *et al*. (2009) and Kois *et al*. (2009)
*D. radiodurans* Smc	RM 30 °C	Discrete foci and 1–3 bright foci per cell	Outer edge of the nucleoid, not regularly positioned	Bouthier de la Tour *et al*. (2009)

MM, minimal medium; RM, rich medium.

These studies using relatively long capture times in epifluorescence microscopy could not reveal the proportion or action of nonfocus molecules at other positions of the chromosome. Nevertheless, the simplest interpretation of the *ori* association is that at least some of the *E. coli* MukBEF action is local at *ori*. In *B. subtilis* and *Streptococcus pneumoniae*, ParB (Spo0J) targets SMC to multiple *parS* sites in the *ori* region, although intriguingly, *B. subtilis* ParB mutants have much weaker phenotypes with respect to chromosome segregation than SMC mutants (Gruber & Errington, 2009; Sullivan *et al*., 2009; Minnen *et al*., 2011), indicating that an alternative mechanism must be available to target the SMC complex to chromosomes or that disruption of *ori* localization does not lead to an SMC^−^ phenotype. In this respect, it is noteworthy that in general, SMC complexes reside at specific regions of chromosomes, although they themselves do not exhibit sequence-specific DNA binding. In *C. crescentus*, no evidence was found for an SMC–ParB interaction (Table 2; Schwartz & Shapiro, 2011). What targets *E. coli* MukBEF complexes to the *ori* region is unknown, but the association requires MukEF and the ability to hydrolyse ATP. Functional association of eukaryotic cohesin with chromosomes has the same requirements (Hu *et al*., 2011). High-resolution quantitative imaging showed that a single *ori*-associated *E. coli* MukBEF focus contains 10 immobile dimer of dimer complexes, on average. These were shown to have a dwell time of *c*. 50 s, while in *B. subtilis*, a somewhat slower turnover rate was calculated (Borgmann *et al*., 2013a). These *in vivo* turnover rates are slower than one might have expected based on *in vitro* ATPase activities, leading to the proposal of a ‘rock climber’ model (above; Badrinarayanan *et al*., 2012b).

Does the *ori* region position SMC or vice versa? The observation that *ori*s are mispositioned in Muk^−^ cells points to MukBEF positioning *ori*s. This view is strengthened by repletion experiments, which indicated that an *ori*-independent MukBEF focus positioning system operates (Badrinarayanan *et al*., 2012a). If this is indeed the case, it indicates that *ori* positioning by MukBEF is central to its role in chromosome segregation. Revealing the mechanism that positions MukBEF will be crucial to understanding the roles of SMC in chromosome segregation.

Mutant MukB proteins that fail to bind ATP or bind ATP, but are defective in head engagement, failed to form Muk fluorescent foci. In contrast, a mutant MukB that binds ATP but is impaired in hydrolysis (MukB_EQ_) has a classical Muk^−^ phenotype, but forms fluorescent foci that do not turnover (Badrinarayanan *et al*., 2012b), consistent with the view that ATP binding and consequent head engagement are necessary for stable loading onto DNA. Intriguingly, fluorescent foci containing this mutant protein had an altered chromosome location (Badrinarayanan *et al*., 2012b; D.J. Sherratt, unpublished data), leading to the conclusion that although ATP binding is sufficient for DNA association, ATP hydrolysis is required for dissociation from DNA and for normal chromosomal location and function. An equivalent mutation in cohesin gave an SMC complex that loaded efficiently at centromeres, but could not establish cohesion or relocate to the normal cohesin binding sites on chromosomes (Hu *et al*., 2011).

The rapidly diffusing *E. coli* MukBEF molecules, which constitute the majority population, appear to have MukB, MukE and MukF complexed together, because they exhibited the same low diffusion coefficient (Badrinarayanan *et al*., 2012b). This contrasts with the conclusion from experiments in *B. subtilis* where the majority diffusing population appeared not to have ScpAB complexed with SMC (Borgmann *et al*., 2013b). Whether these rapidly diffusing molecules are physiologically active in global chromosome organization is unclear; the results with *B. subtilis* would suggest not, at least for this organism. If MukBEF and *ori*-associated bacterial SMC complexes have a direct role in global chromosome organization, then either the non-*ori*-associated molecules must have a role in this or those associated stably with the *ori* must transiently interact with (or ‘gather in’) other regions of the chromosome. In our view, this latter scenario seems unlikely. Alternatively, the primary roles of MukBEF and *ori-*associated SMC complexes may be to position *ori* regions, thereby facilitating bipolar segregation. A failure to do this could indirectly lead to phenotypes of chromosome disorganization and decondensation.

## Concluding remarks and perspective

The remarkable conservation of the architecture of different SMC complexes leads us to be confident that all such complexes share a common biochemical mechanism during their association with, and action on, chromosomal DNA. In our view, the available data support a model in which functional SMC complexes have DNA within the ring formed by the association of SMC with kleisin proteins (Fig. 3). Loading of DNA requires ATP binding and head engagement, with the possibility that DNA is loaded through the dimerization hinge, which may open on head engagement. Loaded DNA may also interact with specific sequences in the SMC, leading to closure of the entrance gate. Given that SMCs are dimeric and cohesin entraps two DNA duplexes, it seems likely that all SMCs will load two DNA segments each interacting with a specific SMC region. Asymmetry in at least the head-engaged complexes appears to be universal for reasons that need to be determined, although it possibly relates to regulation of DNA release from the head exit gate during the ATP binding–hydrolysis–release cycles. The observation that MukBEF dimer of dimer complexes appear to be physiologically relevant, points to mechanisms in which release of DNA from one dimeric complex allows the other dimer of a complex to remain associated with DNA (Badrinarayanan *et al*., 2012b). The likely functional association of MukBEF with TopoIV hints at coordination of decatenation with segregation, two likely sequential steps in the segregation process. Indeed, just as cohesin provides the tension between sister chromatids that is vital for sensing their amphitelic attachment to opposing spindles during successful eukaryotic chromosome segregation, catenation between newly replicated bacterial chromosomes may achieve the same cohesion-like role, with separating *ori*-associated sister MukBEF clusters loaded onto separate sisters fulfilling the role of the eukaryote spindle (Fig. 3d).

Future experiments aimed at revealing the molecular action of SMC complexes will require the multidisciplinary use of structural biology, biochemistry, genetics and cell biology, with an increasing focus on the quantitative live cell imaging methods that will enable real-time *in vivo* biochemistry within the paradigm of ‘observe–measure–perturb’.
